# Intake and food sources of sodium in the population residing in urban areas of Ecuador: results from ELANS study

**DOI:** 10.1080/16549716.2022.2156110

**Published:** 2023-01-05

**Authors:** Mónica Villar, Martha Cecilia Yépez García, María Belén Ocampo, Georgina Gómez

**Affiliations:** a Colegio de Ciencias de la Salud, Universidad San Francisco de Quito, Quito, Ecuador; b Department of Biochemistry, School of Medicine, Universidad de Costa Rica, San José, Costa Rica

**Keywords:** Sodium intake, sodium food sources, sociodemographic characteristics, urban population, ELANS

## Abstract

**Background:**

In 2021, WHO notes that globally, 32% of annual deaths worldwide are due to cardiovascular causes, which have been attributed to excessive sodium intake, and therefore recommends a reduction in salt intake to less than 5 g/day. Ecuador does not have data on sodium consumption in the population. Hence, this study sought to determine the association between sodium consumption and sociodemographic variables in subjects living in urban areas of Ecuador.

**Objectives:**

Determine the main dietary sources of sodium in subjects living in urban areas of the Coast and Highlands of Ecuador, and the association between sodium intake and sociodemographic variables such as: sex, region, marital status, socio-economic and educational level of this population.

**Methods:**

Sodium intake was studied in 800 subjects of both sexes aged 15 to 65 years living in urban areas in Ecuador, originating from the Latin American Nutrition and Health Study (ELANS) between 2014 and 2015. Data were obtained through two 24-hour recalls, and were accessed according to sex, region, age, marital status, socio-economic and educational levels.

**Results:**

The mean sodium intake was 4900 mg/day (SD ± 1188.32 mg/day), and both sexes exceeded the recommendations. Adjusting for energy intake, sodium consumption is higher in participants aged 50–65 years, from low socio-economic status and with basic education level. A positive relationship was found between sodium and energy intake. Around 48% of the sodium sources included the spices, condiments and herbs group. Within this group, salt itself constitutes 99% of sodium sources.

**Conclusions:**

The Ecuadorian population consumes more than double the sodium recommendations, which vary according to gender and age. The first source of sodium is salt itself, which is part of spices and condiments food group. This data is important to formulate public health policies and interventions in Ecuador, especially in the population at risk.

## Introduction

Dietary sodium is necessary for many physiological functions in our body. However, there is also evidence that long-term high sodium intake (measured by urinary excretion) is associated with increased uric acid and urinary albumin excretion. These are markers of endothelial dysfunction that cause harmful effects on blood pressure and lead to an increased arterial hypertension risk [1]. Arterial hypertension, high blood pressure, is the most important risk factor for premature mortality worldwide and a major risk factor for CVD and kidney disease [2]. High sodium intake has been linked to various chronic non-communicable diseases (NCDs) such as hypertension and cardiovascular diseases. These are the leading causes of morbidity and mortality worldwide [3].

For 2021, the World Health Organization (WHO) points out that 32% of annual deaths worldwide were due to cardiovascular causes, which have been attributed to excessive sodium intake. At the same time, excessive sodium intake is one of the major risk factors for CVD. Hence, it is recommended to reduce salt intake to less than 5 g/day and sodium intake of no more than 2000 mg/day to decrease blood pressure and the risk of coronary heart disease and stroke [3].

As a result, various strategies have been implemented in the Americas focusing on reducing salt and sodium consumption by a 30%, goal proposed by WHO by 2025 [4,5]. Strategies include government policies (social development, public health, nutrition), legislation or regulation (nutrition labelling, taxes) and strategies that are part of WHO’s ‘best investments’ (more cost-effective strategies to control food-related chronic non-communicable diseases) [6].

In Ecuador, the Health, and Nutrition Survey (ENSANUT) reported that the prevalence of arterial hypertension in the population aged 18 to 59 years is 9.3% and prehypertension is 37.2% [7]. Despite these figures, few studies show high sodium intake in the Ecuadorian population and very little evidence on sodium intake and sociodemographic correlations [8].

For this reason, this study sought to determine the food sources of sodium in subjects living in urban areas of the Coast and Highlands of Ecuador, and the association between sodium intake and sociodemographic variables such as: sex, region, marital status, socio-economic and educational levels of this population.

## Materials and methods

The Latin American Nutrition and Health Study (ELANS) is a multicenter, cross-sectional study conducted in eight Latin American countries: Argentina, Brazil, Chile, Colombia, Costa Rica, Ecuador, Perú and Venezuela, between 2014 and 2015. The ELANS study was approved by Western’s Institutional Review Board (# 20140605) and was registered in Clinical Trials (# NCT02226627). The study focused on analyzing dietary intake, energy expenditure, and their association with nutritional status. The design involved a multistage complex random sampling, stratified by geographic location, gender, age (15–65 years) and socio-economic status (SES), with a random selection of Primary Sampling Units (PSU) and Secondary Sampling Units (SSU). Selection of respondents within a household was done using 50% of the sample next birthday, 50% last birthday, controlling quotas for gender, age, and SES in order to complete a representative sample. Sample size was calculated with a confidence level of 95% and a maximum error of 3.49. In Ecuador, as in other countries, sample weighting was applied accounting for key variables of interest; the sample consisted of 800 subjects of both sexes, between 15 and 65 years old, residing in urban areas of the main cities related to the country´s population weight of the Coast and Highlands [9].

Written informed consent/assent was obtained from all individuals prior to the start of the study. Pregnant and/or lactating women (in the first 6 months postpartum), individuals with significant physical or mental disabilities that affect food intake or physical activity, individuals outside the age range, adults who did not agree to sign informed consent/assent in the case of minors were excluded from the study.

The stratification of socio-economic level was evaluated using a questionnaire designed by the Ecuadorian National Institute of Statistics and Census (INEC) [10]. Moreover, for purposes of this study it was grouped into three levels: High (A and B), Medium (C +) and Low (C- and D). The questionnaire collected information on essential demographic and socio-economic variables, such as sex (male and female); age group (adolescents [15–18 years], (young adults [19–34 years], adults [35–49 years] and older adults [50–65 years]); educational level (none and basic, high school or bachelor’s degree); marital status (single, marriage, divorce and widowed) and socio-economic level (SEL) (low, mediumor high).

Dietary intake was assessed by two 24-hour recall questionnaires (R24 h), on non-consecutive days, using the Multi-Step Method [11], on weekdays and weekends [12], to estimate routine food consumption and to evaluate intra-individual variability in nutrient intake. A visual guide was used to determine portions and foods to help the participant reference their intake amount. Each R24 h was analyzed, with the Nutrition Data System for Research (NDS-R), Software 2013, developed by the University of Minnesota. A pre-standardization of 220 foods and 130 local recipes was performed to use the NDS-R [12]. Since the nutritional database used in the NDS-R is not specific to Ecuador, the nutritional content of local foods and the database was adapted using the Food Composition Tables of Mexico, Colombia and Perú [13–15].

To obtain greater precision of sodium content in the diet, the following aspects related to R24 h method were considered: sodium from restaurant preparations (through standardized recipes), the amount of sodium in house preparations and the addition of salt in each dish served. Total sodium intake results were adjusted by the Multiple Source Method (MSM) [16].

The analysis of total sodium intake comprised the intrinsic sodium in naturally occurring foods and that which was added during preparation. It was estimated in milligrams of sodium using the MSM software, an online tool that estimates usual nutrient intakes [17].

For the analysis of food sources of sodium, a total of 984 different foods and beverages reported were initially grouped into 96 food groups according to their nutritional characteristics. From these groups, we excluded those that were not considered sodium sources (all these groups together accounted for less than 4% of total sodium intake). The remainder 43 groups were later condensed into a shorter list of 10 food groups according to nutritional similarities or industrial processing (Spices, condiments, and herbs; Rice; Bread, refined grains; Meats, not processed; Cheese; Meats, processed; Soups; Cookies, crackers, and snacks; Diary products; Butter & margarine).

The percentual contribution of each food group to total sodium intake was calculated using the following formula: % sodium from specific food group= (sum of sodium mg from food group/total sum of sodium from all groups) x 100.

Frequency and percentage were used to describe the sociodemographic characteristics of the sample. Descriptive analyses for sodium intake (mg/d) and sodium intake adjusted for 1,000kcal (mg/1000kcal) are presented using arithmetic mean and standard deviation, minimum and maximum sodium intake, stratified by gender, region, age group, educational level, marital status and socio-economic level. Pearson´s correlation coefficients were used to determine the association between sodium and energy intake. Significance was established at a p-value <0.05. Chi-square tests were performed to evaluate the association between categorical variables, and ANOVA to evaluate the association between continuous variables. All analyses were performed using SPSS software (version 22.0; SPSS Inc, Chicago, IL, USA).

## Results

The total sample consisted of 800 subjects between 15 and 65 years. Males and females were represented in practically equal proportions (49.6% and 50.4% respectively). Most of the participants (55%) came from the coastal region. Fifty-two percent (52%) of the respondents were married (or similar), while the rest were single, widowed or divorced. Three-quarters of the group belong to the middle class. The majority have attained basic education (82.8%).

Table 1 shows the sodium intake (mg), according to sociodemographic characteristics, which was on average 4900 mg/day (SD ± 1188.32 mg/day), which compared to the general recommendation of 2000 mg sodium/day [18], most of the participants had sodium intake above this value. Sodium intake in men was significantly higher than that of women (5354.18 mg ± 1122.05 mg v/s 4464.51 mg ± 1086.56 mg) [3].

**Table 1.** Sodium intake by sociodemographic characteristics in the urban Ecuadorian population. ELANS 2014–2015.

	N %	Average	SD	minimum	maximum	p
Sodium (mg)	800 (100)	4901.55	1188.33	2293.79	9978.04	
**Sex**						
Male	397 (49.6)	5345.19	1122.05	2565.73	9978.04	<0.001
Female	403 (50.4)	4464.52	1086.7	2293.79	9317.16	
**Region**						
Coast	360 (45)	4846.76	1254.75	2293.79	9978.04	0.238
Highlands	440 (55)	4946.37	1130.56	2744.24	9317.16	
**Age group (years)**						
15 - 19	128 (16.0)	4867.29	1103.34	2473.03	9317.16	<0.001
20 - 34	316 (39.5)	5100.92	1111.03	2731.83	9978.04	
35 - 49	222 (27.8)	4848.17	1275.61	2293.79	8716.35	
50 - 65	134 (16.8)	4552.56	1212.63	2588.63	9053.01	
**Education level**						
Basic education	664 (83.0)	4858.53	1151.68	2293.79	9317.16	0.077
High school	84 (10.5)	5102.43	1342.12	2342.71	9978.04	
Bachelor Degree	52 (6.5)	5126.41	1344.18	2773.26	8740.19	
**Marital status**						
Single	315 (39.4)	5016.62	1186.76	2473.03	9978.04	0.006
Marriage	414 (51.8)	4882.66	1183.64	2293.79	9053.01	
Divorced	59 (7.4)	4573.91	1164.30	2346.68	6763.91	
Widowed	12 (1.5)	4143.46	1011.57	2832.68	5730.43	
**Socio-economic level**						
Low	399 (49.9)	4917.20	1183.01	2346.68	9317.16	0.447
Medium	297 (37.1)	4928.63	1168.07	2293.79	9978.04	
High	104 (13.0)	4764.18	1266.22	2588.63	9053.01	

Standard Deviation (SD). The value of p is the result of the t-test or ANOVA test.

The age group between 20 and 34 years presented the highest sodium intake (5100.92 ± 1111.03 mg/d), showing significant differences with the group aged 50 to 65 years (*p* < .05). Significant difference was observed between single and divorced people according to marital status.

When studying participants’ energy consumption, the average consumption of 2213.51 ± 601.90 calories/day was observed (Table 2). A relationship between energy and sodium intake was sought, finding a positive and significant correlation between energy intake and sodium intake (r = 0.755, *p* < .001). When sodium intake was adjusted for energy intake, we found an average sodium intake of 2257.98 ± 381.05 mg per 1000 calories consumed. Moreover, when comparing by sex, no differences were found. However, significant differences were found according to the region (highlands 2218.30 v/s coast 2289.63 mg, *p* < .05); educational level (elementary school 2274.69, middle school 2196.26 and high school 2144.32 mg/day, *p* < .05), where the differences are found between basic and higher education levels; and according to socio-economic level, where the participants of the low stratum consume more sodium (2307.04 ± 393.53 mg/day) when compared to those in the high and medium levels (*p* < .05).

**Table 2.** Sodium intake (mg/d) adjusted per 1000 calories by sociodemographic characteristics in the urban Ecuadorian population. ELANS 2014–2015.

	n	Average	SD	Minimum	Maximum	p
Energy (Kcal)	800	2213.51	601.90	824.72	6945.39	
Sodium (mg)/1000Kcal	800	2257.98	381.05	1091.29	4052.38	
**Sex**						
Male	397	2242.52	364.15	1091.29	3978.81	0.225
Female	403	2273.21	396.85	1278.10	4052.38	
**Region**						
Highlands	360	2219.30	404.07	1211.85	3978.81	0.009
Coast	440	2289.63	358.49	1091.29	4052.38	
**Age group (years)**						
15-19	128	2186.45	351.99	1268.31	3363.96	0.001
20-34	316	2225.13	350.07	1091.29	3397.73	
35-49	222	2284.36	406.02	1211.85	4052.38	
50-65	134	2360.10	413.08	1345.97	3978.81	
**Education level**						
Basic education	664	2274.69	378.59	1091.29	4052.38	0.017
High School	84	2196.26	384.17	1436.64	3437.72	
Bachelor Degree	52	2144.32	384.96	1278.10	3008.39	
**Marital Status**						
Single	315	2214.41	369.88	1268.31	3978.81	0.059
Married	414	2286.18	390.73	1091.29	4052.38	
Divorced	59	2303.42	351.68	1497.91	3391.01	
Widowed	12	2205.65	398.51	1655.93	3156.48	
**Socio-economic level**						
Low	399	2307.04	393.53	1278.10	4052.38	0.001
Medium	297	2221.68	357.38	1091.29	3978.81	
High	104	2173.45	374.88	1345.97	3437.72	

Standard Deviation (SD). The value of p is the result of the t-test or ANOVA test.

According to age groups, when adjusted for energy intake, the oldest age group (50–65 years) reported the highest sodium consumption (2360.10 ± 413.08), compared to participants 15 to 19 and 20 to 34 years old (*p* < .05).

**Figure 1.**
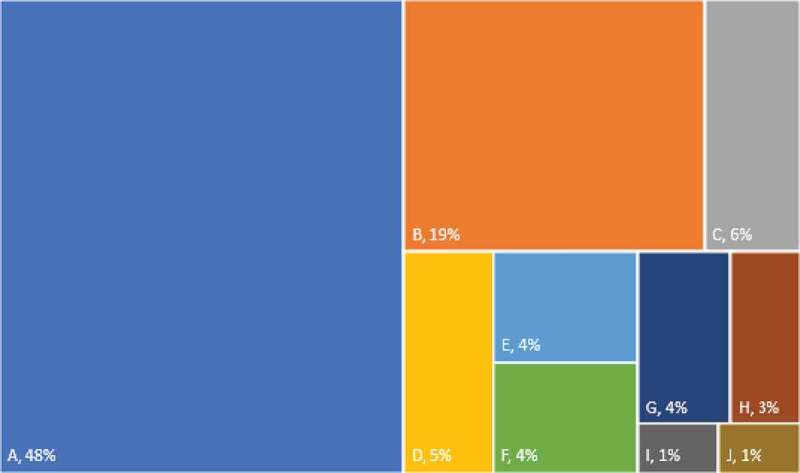
Percentage of sodium contribution from food groups in Ecuadorian population. (A. Spices, condiments & herbs; B. Rice, white; C. Breads, Refined Grains; D. Meat not processed; E Cheese; E. Meat processed; F. Breads, Cookies, crackers & snacks; G. Dairy; H. Butter & margarine.).

The top 10 food sources of sodium are presented in Figure 1. The most common source of sodium, representing 48% of the sodium intake, was the spices, condiments and herbs group. Within this group salt itself constitutes 99%. Rice, prevalent food in Ecuadorian cuisine, accounted for 19% of total sodium intake. At the same time, bread, non-processed meats, cheeses and processed meats had a similar contribution to total sodium intake (6.0%–4.4%).

## Discussion

This study was carried out to show whether sodium intake in the urban Ecuadorian population is within the WHO [3] recommended ranges, and its relation to sociodemographic variables, considering that excessive sodium consumption is one of the main causes of cardiovascular disease development, which is the main cause of morbidity and mortality in Latin America [19]. The WHO indicates that 11 million deaths worldwide are associated with poor diet, of which 3 million are attributed to high sodium intake. All this leads to cardiovascular diseases, the leading cause of deaths worldwide. Therefore, reducing sodium in the diet is recommended, taking into account that this component does not only consider table salt, but several food products as well [3]. On the other hand, it should be noted that reducing sodium intake is beneficial in both hypertensive and normotensive individuals [20].

The gold standard for measuring sodium intake is 24-hour urinary excretion of the mineral [21]; however, this method involves greater difficulty and a higher cost. Furthermore, the Dietary Reference Intakes for Sodium and Potassium 2018, and evidence from available studies, suggest that urinary sodium excretion does not reflect short-term sodium intake, reducing the method´s validity [22]. When using 24-hour recall questionnaires, it is recommended that more than one survey be collected on both weekdays and weekends, and estimate habitual intake using statistical methods, performed in the ELANS study [12]. For this reason, most population studies, especially in Latin America, apply other methodologies such as evaluating of dietary intake under a protocol that allows obtaining greater accuracy in the results [19,20,22].

This study´s results showed that the average sodium intake was 4901 mg/d (±1118.33 mg), a value that doubles the amount of sodium the World Health Organization recommends for daily intake [3]. Different results have been found in different regions and continents around the world. In Latin America, high values of salt consumption can be seen, ranging between 7 and 13 grams of salt/day, equivalent to approximately 2800 to 5200 mg sodium/day [19,20]. In Argentina, 4700 milligrams/day of consumption has been reported, which exceeds the established recommendations [23]. A multicenter study conducted in Latin America (Chile, Peru, Ecuador, Honduras and Paraguay) also recorded a sodium intake above WHO recommendations, on average ranging between 4525 mg/day and 6689 mg/day [24]. All of them were measured using R24 h. The most recent study in Ecuador measuring sodium excretion in urine shows a consumption of 2 655(±1 185); however, this pilot study may present a bias since the population studied was university and health personnel of a teaching hospital and the education level may influence the higher or lower sodium consumption [8]. At the national level, data from the STEPS ECUADOR 2018 Survey [25] shows that the population does take actions to reduce foods rich in salt through the review of food labeling, or avoiding processed foods, but still maintains the habit of putting salt on food during preparation or in the dish served [25].

Regarding sex, the mean values of sodium consumption were significantly higher in men (5387.4 mg/day) than in women (4423.7 mg/day). These results could be compared with those found in several Latin American countries such as Brazil [26,27], Uruguay [28] and Chile [29]. As in this study, the included population exceeds the recommendations, and men have higher consumption than women. However, this is mainly due to the higher caloric intake of men due to higher expenditure than women. When analyzing the sodium intake adjusted for energy, it could be corroborated that both sexes have similar consumption without significant differences. Similar data was obtained by the Public Health England 2014 report [30].

Considering the usual sodium intake according to age groups, young adults (20 to 34.9 years) have the highest sodium intake among the other age groups, with an average intake of 5100.92 mg ± 1111.03 mg per day. Older adults (50 to 65 years) consume the least sodium per day, with an average of 4552.5 mg (±1212.55 mg), with differences being found between these two groups. This data is similar to that reported by Pereira, et al. 2019, where a higher sodium consumption was obtained in people within the age range of 45 to 65 years, evidencing a significant association (27) and different from other studies on the Uruguayan population. Although the age of the participants was within the range of 34 to 74 years, found no difference between the groups [2]. However, when adjusting sodium by the energy consumed, the group of older adults (50 to 65 years) showed the highest sodium consumption, with significant differences among all other age groups. This data allows us to conclude that, when adjusting for energy, older individuals have a lower-diet quality than younger ones, which is not evident since caloric intake is lower.

According to Elliot and Brown (2007), the values found may be due to physical activity according to age ranges. People between 20 and 35 years of age are more physically active and therefore tend to consume more food. Similarly, economically active people in these ranges tend to eat more fast food and food cooked outside the home. Thus, it seems that sodium intake decreases after the age of 50, but it is proportional to energy consumption [31].

Regarding socio-economic and educational levels, differences were only seen when adjusting for energy. Participants with a low socio-economic level and basic education had a higher sodium intake. This may be caused by the food´s quality (due to lower cost) and the use of salt in the preparations. Pereira 2019 found similar results showing the consumption of foods with high sodium content, such as lower-cost processed and ultra-processed products, and the addition of table salt and industrialized seasonings when preparing meals [27]. Other studies have also found relationships between high sodium intake and socio-economic status, similar to our data [2].

Among the food groups that contribute the most to sodium intake are spices, condiments and salt (48.1% contribution), which indicates that added salt contributes the most to the high daily sodium intake. The next food group that contributes sodium is rice, which can be considered as a traditional food in the country and has already been reported in the National Health and Nutrition Survey 2014 as the main contributor of sodium and calories in the Ecuadorian population [7]. In a lower percentage but with similar values are bread, refined grains, meats (unprocessed) and eggs, cheese, processed meats, soups, crackers, crackers and snacks. Similarly, a study conducted in Brazil [32] found that the foods with the highest amount of sodium were dressings, meat products and bread; as well as in Colombia [19], where the main source of sodium intake was bakery products, and below them were sausages, dressings, traditional foods, cheeses and snacks. However, it is important to consider that, according to the 2005 DRIs for sodium, the main sources of sodium in the diet are: sodium added to foods during processing or cooking and the native content of this mineral in natural foods. Furthermore, regardless of its origin, it is estimated that 90% of the dietary sodium ingested is found as sodium chloride (known as table salt) [33].

Finally, the present study has several strengths, provides information on sodium intake in the Ecuadorian population living in urban areas in relation to energy intake and its food sources, the application of two 24-hour recall questionnaires (R24 h), on non-consecutive days following a standardized methodology, also that the interviews considered individually the salt added to meals, unlike other studies. Although the present study has some limitations, the data represent the dietary and sodium intake of the urban population of Ecuador and caution should be used to extrapolate these findings to other countries. Regarding the selected traditional methodology used to evaluate sodium intake, it is necessary to recognize that dietary intake evaluation is subject to random and systematic errors, despite the care taken to minimize them, as presented elsewhere [12]. Furthermore, the data found could contribute to the creation of health policies and interventions on sodium consumption at the national level, especially in the population at risk, considering that sodium intake exceeds twice the recommendations, and that ischemic heart disease is the first cause of death in the country [34].

In addition, because the salt added to food (table salt and condiments) is the main source of sodium in the diet, healthy eating strategies should prioritize the rescue of traditional cuisine, highlighting the messages of the dietary guidelines for the Ecuadorian population that are based on food [35] and including messages of reduction of the use of salt and salt substitutes. On the other hand, industrialized foods collectively represent more than 50% of the sodium in the diet. Thus, programs or policies focused on reducing sodium intake can be implemented such as switching to front labeling of packaged foods, modifying the maximum sodium content in foods or restrictions on salty foods for children.

Therefore, it is necessary to continue investigating how to motivate the reduction of salt intake and strengthen the promotion of healthy eating, rescuing the traditional cuisine based on natural foods, and exploring why individuals continue to lead an unhealthy lifestyle.

## Conclusions

The Ecuadorian population consumes more than double the sodium recommendations, which vary by sex and age when calorie intake is adjusted. The first source of sodium is the salt itself which is part of the group of spices and condiments. This study provides valuable information on sodium consumption in the country, considering that there is no published data for this nutrient at the population level, which is necessary to formulate public health policies and interventions to be implemented in Ecuador, especially in the population at risk, taking into account the high sodium intake, and that ischemic heart disease is the leading cause of death in the country [31]. Therefore, it is necessary to continue investigating how to motivate the reduction of salt intake and strengthen the promotion of healthy eating, rescuing the traditional cuisine based on natural foods and exploring why individuals continue to lead unhealthy lifestyles.

## Paper context

Dietary sodium plays a role in the development of cardiovascular diseases and premature mortality worldwide. This study determines the association between sodium consumption and sociodemographic variables in urban areas of Ecuador. The Ecuadorian population consumes almost two times the recommended sodium. Salt added to meals is the main source of sodium in the diet. Thus, it is necessary to motivate the reduction of salt intake and promote healthy eating and lifestyles.
